# Gut microbiome response to a modern Paleolithic diet in a Western lifestyle context

**DOI:** 10.1371/journal.pone.0220619

**Published:** 2019-08-08

**Authors:** Monica Barone, Silvia Turroni, Simone Rampelli, Matteo Soverini, Federica D’Amico, Elena Biagi, Patrizia Brigidi, Emidio Troiani, Marco Candela

**Affiliations:** 1 Unit of Microbial Ecology of Health, Department of Pharmacy and Biotechnology, University of Bologna, Bologna, Italy; 2 Primary Care Unit and Territorial Health, Social Security Institute, Cailungo, Republic of San Marino; University of Illinois, UNITED STATES

## Abstract

The modern Paleolithic diet (MPD), featured by the consumption of vegetables, fruit, nuts, seeds, eggs, fish and lean meat, while excluding grains, dairy products, salt and refined sugar, has gained substantial public attention in recent years because of its potential multiple health benefits. However, to date little is known about the actual impact of this dietary pattern on the gut microbiome (GM) and its implications for human health. In the current scenario where Western diets, low in fiber while rich in industrialized and processed foods, are considered one of the leading causes of maladaptive GM changes along human evolution, likely contributing to the increasing incidence of chronic non-communicable diseases, we hypothesize that the MPD could modulate the Western GM towards a more “ancestral” configuration. In an attempt to shed light on this, here we profiled the GM structure of urban Italian subjects adhering to the MPD, and compared data with other urban Italians following a Mediterranean Diet (MD), as well as worldwide traditional hunter-gatherer populations from previous publications. Notwithstanding a strong geography effect on the GM structure, our results show an unexpectedly high degree of biodiversity in MPD subjects, which well approximates that of traditional populations. The GM of MPD individuals also shows some peculiarities, including a high relative abundance of bile-tolerant and fat-loving microorganisms. The consumption of plant-based foods–albeit with the exclusion of grains and pulses–along with the minimization of the intake of processed foods, both hallmarks of the MPD, could therefore contribute to partially rewild the GM but caution should be taken in adhering to this dietary pattern in the long term.

## Introduction

In order to understand the specificities of the human microbiome assembly, extensive meta-analyses of human and non-human primate microbiomes have been recently carried out [1,2]. This comparative approach has led to the identification of several compositional changes along with a progressive reduction of biodiversity as the main distinctive features of the human gut microbiome (GM) along the evolutionary history [1]. Interestingly, these hallmarks have been found to be exacerbated in Western urban populations compared to traditional and rural counterparts [3–6]. In particular, consistent with the disappearing microbiota hypothesis [7], the dramatic shrinkage of individual GM diversity in Western urban populations is deemed to depict a maladaptive microbiome state which may contribute to the rising incidence of chronic non-communicable diseases, such as obesity, diabetes, asthma and inflammatory bowel disease [8–11]. Consequently, in recent years, a large body of research has been devoted to understanding the mechanisms leading to the alterations in the Western urban GM. It is in this scenario that the multiple-hit hypothesis has been advanced [8]. According to this theory, the progressive changes in the human GM and especially the reduction of biodiversity have occurred at multiple stages along the recent transition to modern urban societies, and several aspects typical of the urbanization process—such as sanitation, antibiotics, C-section and Western diet—have been pointed out as contributing factors. In particular, the reduction in quantity and diversity of Microbiota-Accessible Carbohydrates (MACs) in the diet has been considered one of the leading causes of the disappearing GM in Western urban populations [8]. Recently defined, dietary MACs include all types of carbohydrates, coming from a variety of sources including plants, animal tissue or food-borne microbes, which—indigestible by the host—become available as an energy source for a specific GM fraction enriched in Carbohydrate Active Enzymes (CAZymes) [8,12]. Moreover, food additives, emulsifiers and xenobiotics–ubiquitous in industrially processed foods–have recently been shown as important additional drivers of GM diversity shrinkage [13].

All currently available studies exploring the disappearing GM are based on the comparison between Western urban and traditional rural populations [3–6,14–16]. Consistently, the observed GM differences are likely to be the result of the combined action of several covariates in addition to the diet–i.e. ethnicity, geographical origin, climate, subsistence, medication, hygiene and life sharing–and do not allow to weight the importance of the single determinants.

In the last few years, the Modern Paleolithic Diet (MPD), with high intake of vegetables, fruit, nuts, seeds, eggs, fish and lean meat, while excluding grains, dairy products, salt and refined sugar, has attracted substantial public attention in the Western world because of its potential multiple health benefits [17–22].

In the present work, we profiled the GM structure of 15 Italian subjects following the MPD and compared it with that of 143 urban Italian individuals largely adhering to the Mediterranean Diet (MD) from our previous works [5,23]. Notwithstanding the small sample size, our GM exploratory study gave us the unique opportunity to assess to what extent the adoption of a Paleolithic dietary pattern, based on the consumption of MACs deriving from plant-based foods–but not grains–along with the exclusion of industrially processed food, may modulate the GM of Western urban populations, possibly helping to counteract the GM diversity reduction. Indeed, the comparison between MPD and Western diets in subjects living in the same country allows excluding the impact of confounding drivers of GM variation, such as geography, ethnicity, medication, hygiene and subsistence [14,15,23]. In order to extend the GM comparison at the meta-population level, we included in our analysis publically available microbiome data from traditional hunting and gathering populations showing an “ancestral” high-diverse GM profile, such as the Hadza from Tanzania, from our previous publication [5], the Matses from Peru [6], and the Inuit from the Canadian Arctic [24].

According to our data, the consumption of unprocessed foods and dietary MACs from plant-based foods–albeit with the exclusion of grains and pulses–as observed in MPD individuals, could contribute to a high GM diversity, similar to that typically found in traditional rural populations, even in a Western urban context. However, we also identified peculiar compositional features, such as a high relative abundance of bile-tolerant and fat-loving microbes, worthy of being further investigated for the potential health risk.

## Results

### Diet, socio-economic context and gut microbiome structure in Italian adults following the modern Paleolithic diet

Fifteen healthy individuals, 12 males and 3 females, who have been following the MPD for at least one year were recruited from different urban areas across Italy. The average age of the enrolled subjects was 39.2 years (range, 26–57), and the average Body Mass Index (BMI) 22.1 kg/m^2^ (range, 19.4–25.7) (S1 Table).

**Fig 1:**
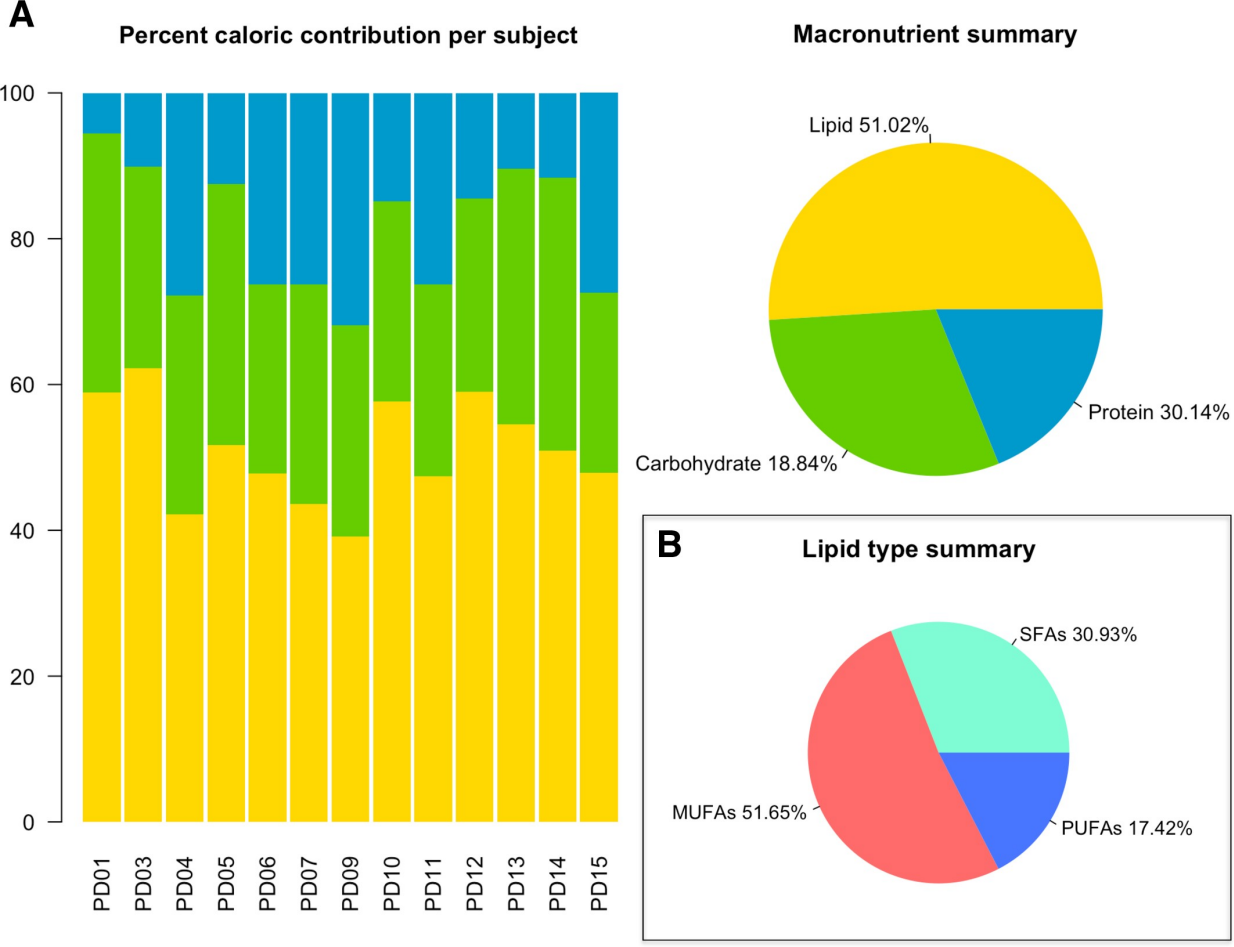
Macronutrient composition of the modern Paleolithic diet. (A) Bar plots of the percent caloric contribution of fat, protein and carbohydrate per subject, based upon weighted food intake records over 7 days. The pie chart shows the summary of the average macronutrient intake for the entire cohort. (B) Pie chart of the lipid type summary. PUFAs: polyunsaturated fatty acids; MUFAs: monounsaturated fatty acids; SFAs: saturated fatty acids.

The MPD adopted by the 15 subjects is mainly based on the consumption of unprocessed foods, with high intake of vegetables, fruit, nuts and seeds, eggs, fish and lean meat, while excluding grains, dairy products, salt and refined sugar. The daily total calorie intake, as well as that of macro- and micro-nutrients, assessed through 7-day weighted food intake records (7D-WRs), are reported in S2 Table. The average daily energy intake of the enrolled cohort is 1,843.45 kcal (range, 1,563–2,186 kcal). The percentage of macronutrients is distributed as follows: fat, 51.02%; protein, 30.14%; carbohydrate, 18.84% (Fig 1A). With regard to lipids, 51.65% of total calories are from monounsaturated fatty acids (MUFAs), 30.93% from saturated fatty acids (SFAs) and 17.42% from polyunsaturated fatty acids (PUFAs) (Fig 1B). The average daily fiber intake is 14.64 g/1,000 kcal.

Based on the data collected through a questionnaire on the socio-economic status, one third of the subjects lived in highly urbanized areas, more than half in semi-urbanized areas (8/15) and only one individual in a rural setting. Two thirds lived in apartments and the remainder in independent houses. Eight out of 15 subjects declared they had pets and daily contact with nature (defined as 2 to 15 hours a week spent in a green area). According to a questionnaire on physical activity (the Global Physical Activity Questionnaire—GPAQ), 12 individuals reported practicing moderate to intense fitness activities for an average of 1 hour a day for at least 3 days a week.

**Fig 2:**
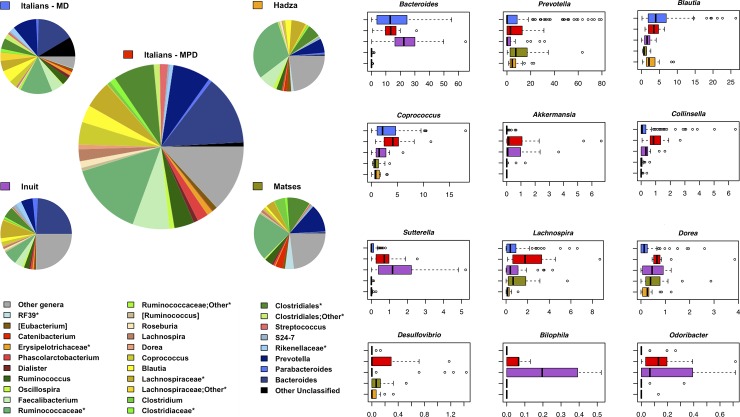
Genus-level phylogenetic structure of the gut microbiome of Italian adults adhering to the modern Paleolithic diet and major differences among study groups. Pie charts show the average relative abundance of bacterial genera represented in the GM of the enrolled study groups (in the center, urban Italians adhering to the modern Paleolithic diet from the present study; on the sides, urban Italians adhering to the Mediterranean diet [23], Hadza from Tanzania [5], Matses from Peru [6], and Inuit from Canadian Arctic [24]). Only bacterial genera with relative abundance > 0.5% are shown. Boxplots show the relative abundance distribution of significantly different bacterial genera among study groups. *, unclassified OTU reported at higher taxonomic level.

The GM structure of MPD Italian adults was profiled through 16S rRNA gene sequencing of fecal DNA. A total of 864,439 high-quality reads (mean ± sd, 57,629 ± 19,752; range, 25,142–95,924) were generated and clustered in 7,483 OTUs. The phyla Firmicutes (relative abundance, mean ± sem, 65.1 ± 2.1%) and Bacteroidetes (24.6 ± 2.2%) dominate the gut microbial ecosystem, with Proteobacteria (4.4 ± 1.6%), Actinobacteria (3.4 ± 0.8%) and Verrucomicrobia (1.2 ± 0.5%) as minor components. At family level, *Ruminococcaceae* (26.7 ± 1.7%), *Lachnospiraceae* (18.7 ± 1.4%), *Bacteroidaceae* (13.7 ± 1.8%) and *Prevotellaceae* (7.4 ± 2.4%) are the dominant GM constituents. The most abundant (≥ 5%) bacterial genera are *Bacteroides*, *Prevotella*, and *Faecalibacterium*, while *Coprococcus*, *Ruminococcus*, *Blautia*, *Lachnospira*, *Phascolarctobacterium*, *Streptococcus*, *Roseburia*, *Akkermansia*, *Oscillospira* and *[Eubacterium]* represent minor components of the microbial ecosystem (range, 4.4 ± 0.7% - 1.0 ± 0.4%) (Fig 2; S1 Fig).

### Gut microbiome diversity in MPD Italian adults and comparison with other Western urban populations and traditional communities

**Fig 3:**
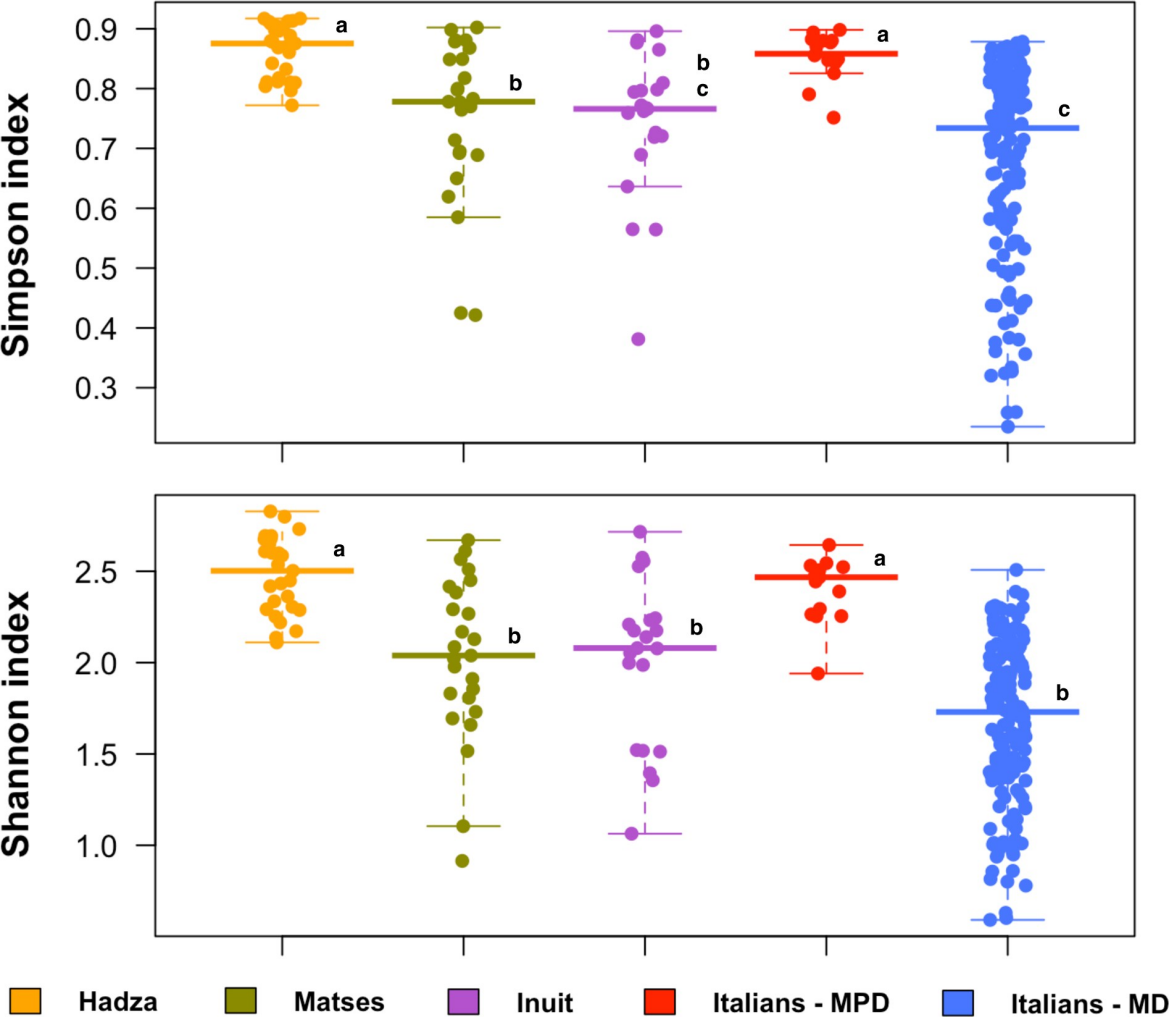
The gut microbiome of Italian subjects following the modern Paleolithic diet shows intermediate biodiversity between Western urban and traditional populations. Box and scatter plots showing the alpha diversity values, measured with Simpson and Shannon indices, for each study population (i.e. urban Italians adhering to the modern Paleolithic diet from the present study, urban Italians adhering to the Mediterranean diet [23], Hadza from Tanzania [5], Matses from Peru [6], and Inuit from Canadian Arctic [24]. Different letters above the median line indicate significantly different groups (P-value < 0.05, Wilcoxon test). MPD = Modern Paleolithic Diet; MD = Mediterranean Diet.

In order to investigate whether the adherence to the MPD is sufficient to promote a more diverse GM ecosystem—even in a Western urban context—we compared the GM diversity between the 15 MPD subjects and 143 urban Italians with different level of adherence to the MD, whose GM composition was described in De Filippis *et al*. (n = 127) [23] and Schnorr *et al*. (n = 16) [5]. Moreover, to extend the comparative analysis to a global level, the GM structural profiles of the following traditional hunter-gatherer populations were included: 27 Hadza from Tanzania [5], 25 Matses from Peru [6], and 21 Inuit from Canada [24]. According to our findings, significant differences in the GM biodiversity among the study groups were detected (Simpson index, P-value = 2.6 × 10^−15^; Shannon index, P-value = 2.2 × 10^−16^; Kruskal-Wallis test) (Fig 3). Interestingly, the GM diversity observed for MPD subjects far exceeds that of urban Italians adhering to the MD (Simpson index, P-value = 2.5 × 10^−7^; Shannon index, P-value = 6.1 × 10^−9^; Wilcoxon test), is comparable to that of the Hadza (P-value = 0.39; 0.26), and even greater than Matses (P-value = 0.0082; 0.0039) and Inuit (P-value = 0.00075; 0.0027).

**Fig 4:**
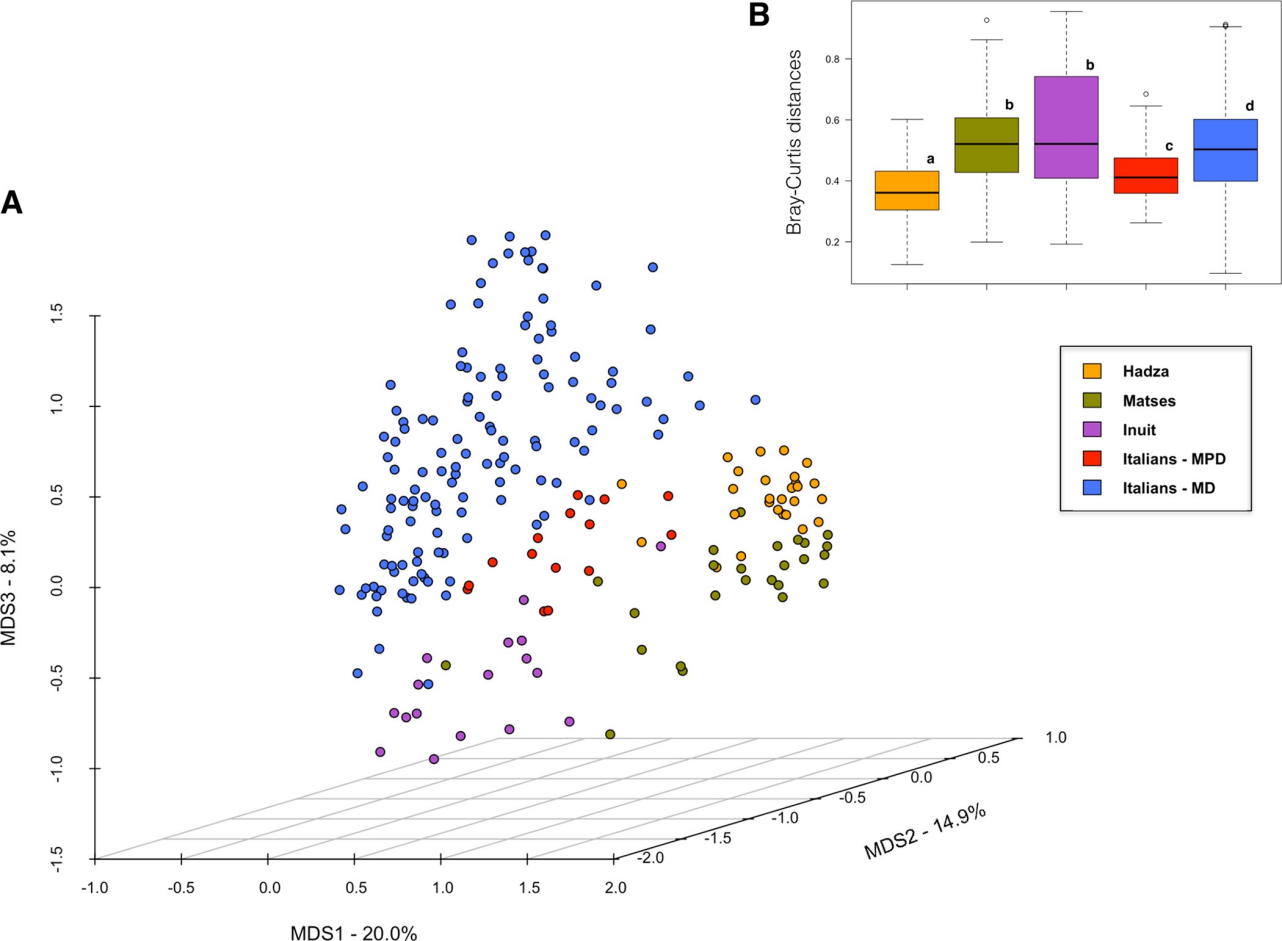
Beta diversity of the fecal microbiome of Italian subjects following the modern Paleolithic diet compared with other Western urban populations and traditional communities. (A) The PCoA plot shows the Bray-Curtis distances between the genus-level microbiota profiles of urban Italians adhering to the modern Paleolithic diet from the present study, urban Italians adhering to the Mediterranean diet [23], Hadza from Tanzania [5], Matses from Peru [6], and Inuit from Canadian Arctic [24]. A significant segregation among study populations was found (P-value < 1 × 10^−5^; permutation test with pseudo-*F* ratios). (B) Boxplots show the interpersonal variation, in terms of Bray-Curtis distances between the genus-level microbiota profiles, for each study group. Different letters in the boxplots indicate significant differences (P-value < 0.05, Wilcoxon test). MPD = Modern Paleolithic Diet; MD = Mediterranean Diet.

The PCoA based on Bray-Curtis distances was next used to assess overall genus-level compositional differences in the GM structure between study groups. Our data show clear separation of GM profiles by provenance and, within the Italian cohort, by dietary pattern (adonis: P-value < 1 × 10^−5^, R^2^ = 0.27; ANOSIM: P-value < 1 × 10^−5^, R = 0.48) (Fig 4A; S3 Table). Interestingly, MPD subjects show a low level of interpersonal GM variation (Bray-Curtis distances, mean ± sd, 0.42 ± 0.095), approximating that observed for the Hadza (0.36 ± 0.092) (Fig 4B). In order to identify the bacterial drivers with a statistically significant contribution (permutation correlation test, P-value < 0.001) to the sample ordination, we superimposed the genus relative abundance on the PCoA plot (S2 Fig). According to our data, the microorganisms characterizing the Italian cohort are *Bacteroides*, *Collinsella*, *Coprococcus* and *Blautia*. The genera *Clostridium*, *Prevotella*, *[Prevotella]*, *Catenibacterium* and *Oscillospira* were found to be associated with Hadza and Matses, while *Sutterella* and *Parabacteroides* with Inuit.

Despite the small sample size, it is worth noting that the MPD microbiome shows several compositional differences with respect to the other cohorts, which well match the peculiar macronutrient intake (Fig 2; S4 Table). In particular, compared with all other populations, except for the Inuit (as expected based on available dietary information), the MPD fecal profiles are enriched in asaccharolytic genera, such as *Sutterella* and *Odoribacter* [25], in *Bilophila*, microorganism typically associated with animal protein and saturated fat consumption [26,27], as well as in *Akkermansia*, known to be associated with the consumption of unsaturated fat [28] (P-value ≤ 0.021; Wilcoxon test). Although *Akkermansia* has recently been identified as potential next-generation probiotics, its role in inflammatory contexts is still controversial and requires further investigation [28–31]. Moreover, when compared to hunter-gatherer populations (whose subsistence, at least during sampling, was mainly based on tubers and other plant foods), the microbiome of MPD subjects shows increased relative abundance of the bile-tolerant *Bacteroides*, *Collinsella* and *Dorea* (P-value ≤ 0.003). *Bacteroides* is indeed typically associated with Western-type animal-based diets [26], the genus *Collinsella* is known to comprise bacterial species capable of deconjugating bile acids and positively correlated with plasma cholesterol levels [32], and *Dorea* has recently been suggested to be involved in the production of the secondary bile acid, deoxycholic acid [33]. It should be remembered that secondary bile acids are generally associated with increased risk of non-infectious bowel disease and colorectal cancer [34], which stresses the need to be cautious in adhering to this dietary pattern in the long term. On the other hand, it should be noted that, compared to traditional populations, MPD profiles show greater proportions of the SCFA producers *Lachnospira* and *Coprococcus* (P-value ≤ 0.008).

We also evaluated the *Prevotella* ratio, i.e. the ratio of *Prevotella* to the sum of *Prevotella* and *Bacteroides* [35] (S3 Fig). These genera are indeed recognized as biomarkers of diet and lifestyle, with *Bacteroides* typically associated with high-protein high-fat Western diets and *Prevotella* with carbohydrate/fiber-based diets typical of more agrarian societies [35,36]. Although no detailed dietary information is available for traditional populations, Hadza and Matses diets are known to be heavily based on the consumption of highly fibrous tubers and vegetal foods [5,6]. On the other hand, the fiber intake of MPD individuals (29.47 ± 20.49 g/day) does not exceed by far that reported for urban MD Italians (range, 10.37–21.02 g/day; [23]). Consistent with this, a significantly lower *Prevotella* ratio was observed for MPD individuals as well as for other urban Italians compared to Hadza and Matses (P-value < 6.6 x 10^−7^).

## Discussion

Herein we compared the GM compositional structure and diversity of urban Italian adults adhering to the MPD with previously published data from urban Italian adults largely adhering to the MD [5,23] and traditional hunter-gatherers, including Hadza from Tanzania [5], Matses from Peru [6], and Inuit from Canada [24].

According to our findings, the microbiomes of the study groups segregate by geographical origin, with a further separation within the Italian cohort reflecting the diet pattern (MPD vs MD). This provenance-dependent effect on the human GM structure probably involves the concomitant action of several covariates, which concur in shaping the GM structure, such as geography, ethnicity, lifestyle and dietary habits. The Italian origin of the GM seems to be defined by a higher abundance of *Bacteroides*, *Collinsella*, *Coprococcus* and *Blautia*, bacterial genera commonly found within Western healthy microbiomes [3–6]. According to the literature, the separation due to geography seems to be less evident among the traditional populations, with Matses and Hadza sharing a high abundance of *Prevotella* [5,6]. These data confirm recent findings that demonstrate the predominance of host location and ethnicity (including diet, lifestyle, environmental exposure, socio-economic development, etc.), with respect to diet alone, as determinants of human GM variation [14,15].

Despite the overall Western-like configuration, the MPD-associated GM structure stands out from that of urban Italians adhering to the MD for several features that could be related to the peculiar dietary pattern. These mainly include a greater relative abundance of asaccharolytic bacteria (i.e. *Sutterella* and *Odoribacter*) [25] as well as of fat- and bile-loving microorganisms, such as *Bilophila* [26,27]. In light of the known associations between changes in the bile acid pool, in particular with increased production of secondary bile acids, and increased risk of non-infectious bowel disease and colorectal cancer [34], the increased presence of these bacteria could constitute a red flag for human health, worthy of being further explored possibly in long-term studies.

On the other hand, it is worth noting that the levels of fiber-degrading SCFA producers, such as *Faecalibacterium*, *Ruminococcus*, *Lachnospira* and *Coprococcus*, are comparable between MPD subjects and other Italians, suggesting that even excluding grains and legumes, the high serves of fruit, vegetables, nuts and seeds in the MPD could ensure adequate supply of MACs to the GM. The most interesting data is, however, the much higher degree of microbiome biodiversity found in MPD individuals than other Italians, which well approximates that observed in traditional hunter-gatherer populations. As recently discussed, a high species diversity could promote healthy competition among microbial symbionts and modulate bacterial interactions, ultimately maintaining the overall ecosystem stability [37]. Our findings therefore seem to suggest that even in extremely different geographic locations, with disparate cultural practices, environmental exposure, economic development and other lifestyle factors, the ancestral microbiome could be at least partly restored. Since the Italian subjects of our cohort share the provenance and all that it entails, including the lifestyle, it can be hypothesized that the MPD-associated bloom in GM diversity is accounted for by the peculiarities of the MPD compared to the MD. Though the two diets are similar in many respects–i.e. high intake of fruit, vegetables, fish and nuts, as well as low glycemic load–the MPD is in fact distinguished by: (i) consumption of MACs from plant foods but excluding grains and legumes; (ii) total exclusion of industrially processed products; (iii) higher intake of unsaturated fatty acids, especially MUFAs, from olive oil, nuts and meat; (iv) no consumption of foods containing refined sugars [17–20]. It is, therefore, tempting to speculate that these MPD distinctive features may be sufficient to support the consolidation of a highly diversified GM layout, thus counteracting the loss of GM biodiversity, typically observed in Western urban populations as compared to traditional communities [3–6]. However, at least two important considerations must be made in relation to biodiversity: i) simplifying the GM to a measure of biodiversity has obvious limitations as it does not reflect its compositional structure, including the complex ecological interactions existing among its members [38]; ii) a reduced diversity is not necessarily detrimental to the host, especially when it is a consequence of the selective enrichment of health-promoting symbionts [37–39].

In conclusion, we shed some light on the effects of the MPD on the GM structure and diversity in Western urban populations. Despite the limitations of this observational study (i.e. cross-sectional nature and small sample size), our findings suggest that the MPD could be a means to counteract the risk of losing the bacterial memory that has accompanied our ancestors throughout human evolutionary history. The consumption of MACs from plant-based foods–but not grains–at the expense of refined sugars, and the minimization of the intake of processed foods, both hallmarks of the MPD, could indeed act synergistically in maintaining an eubiotic level of GM diversity. The high intake of MUFAs, as found in the MPD, suggests that these fatty acids could play a role in supporting high GM diversity, which is worthy of being further explored in larger cohorts. However, we cannot exclude that other genetic or lifestyle-related factors not considered in the present study are involved. On the other hand, we do not know how this high-diverse GM will behave over time in a context so different from that of our ancestors. Furthermore, the presence of some red flags, such as the overrepresentation of bile and fat-loving microbes, requires attention for potential long-term health effects. Albeit several studies have suggested intriguing potential benefits of the MPD in obese and type 2 diabetes patients in the medium and long term (i.e. increase in insulin sensitivity, glycemic control and leptin levels, and lowering of total fat mass and triglyceride levels) [18,40,41], particular caution must be taken when following Paleolithic diets for a long time with percentages of macronutrients so far from nutritional recommendations, at least until more comprehensive longitudinal studies in larger cohorts, including randomized controlled trials, fully assess the MPD impact on host health.

## Materials and methods

### Subjects and sample collection

Fifteen healthy individuals following a MPD for at least one year were recruited from different urban areas across Italy (Lombardia, Piemonte, Emilia-Romagna, Toscana, Umbria, Lazio, Campania, Molise, Puglia and Calabria regions). Exclusion criteria included: age below 18 and over 65 years; BMI <18.5 and >24.9 kg/m^2^; habitual intake of drugs and nutritional and pharmacological supplements of pre- and probiotics; taking antibiotics in the last three months; presence of intestinal and metabolic disorders (i.e. inflammatory bowel disease, bacterial contamination syndrome, irritable bowel syndrome, constipation, celiac disease, type 1 and 2 diabetes, cardio- and neurovascular diseases, rheumatoid arthritis, allergies, neurodegenerative diseases, cancer). Written informed consent was obtained from all volunteers. All work was approved by the Ethics Committee of the Sant’Orsola-Malpighi Hospital, University of Bologna (ref. number, 118/2015/U/Tess).

Each subject was asked to fill in a 7-day weighted food intake record (7D-WR), with the total food and beverage consumption (including methods of preparation whenever possible) for 7 days representing their usual intake, as previously described [42]. Daily total calorie intake as well as that of macro- and micro-nutrients were assessed through the MètaDieta software version 3.7 (METEDA). The participants were also asked to fill in two questionnaires, one regarding their socio-economic status (according to the guidelines of the Health Survey for England– 2013, http://www.hscic.gov.uk/catalogue/PUB16076) and the other on physical activity (based on the Global Physical Activity Questionnaire–GPAQ–developed by World Health Organization, http://www.who.int/chp/steps/resources/GPAQ_Analysis_Guide.pdf). A single fecal sample was self-collected by each participant after completing the 7D-WR (i.e. on day 7) and immediately frozen at -20°C. All specimens were delivered to the laboratory of the Microbial Ecology of Health Unit (Dept. Pharmacy and Biotechnology, University of Bologna, Bologna, Italy) where they were stored at -80°C until processing. Data and fecal samples were collected between March and April 2017.

### Microbial DNA extraction

Total bacterial DNA was extracted from each stool sample using the DNeasy Blood and Tissue kit (QIAGEN) with the modifications previously described by Biagi *et al*. [43]. In brief, 250 mg of fecal samples were suspended in 1 ml of lysis buffer (500 mM NaCl, 50 mM Tris-HCl pH 8, 50 mM EDTA, 4% (w/v) SDS), added with four 3-mm glass beads and 0.5 g of 0.1-mm zirconia beads (BioSpec Products) and homogenized using a FastPrep instrument (MP Biomedicals) with three bead-beating steps at 5.5 movements/sec for 1 min, and 5-min incubation in ice between treatments. After incubation at 95°C for 15 min, stool particles were pelleted by centrifugation at 14,000 rpm for 5 min. Nucleic acids were precipitated by adding 260 μl of 10 M ammonium acetate and one volume of isopropanol. The pellets were then washed with 70% ethanol and suspended in TE buffer. RNA was removed by treatment with 2 μl of DNase-free RNase (10 mg/ml) at 37°C for 15 min. Protein removal and column-based DNA purification were performed following the manufacturer’s instructions (QIAGEN). DNA was quantified with the NanoDrop ND-1000 spectrophotometer (NanoDrop Technologies).

### 16S rRNA gene sequencing

For each sample, the V3-V4 region of the 16S rRNA gene was amplified using the S-D-Bact-0341-b-S-17/S-D-Bact-0785-a-A-21 primers [44] with Illumina overhang adapter sequences. PCR reactions were performed in a final volume of 25 μl, containing 12.5 ng of genomic DNA, 200 nM of each primer, and 2X KAPA HiFi HotStart ReadyMix (Kapa Biosystems, Roche), in a Thermal Cycler T (Biometra GmbH) with the following gradient: 3 min at 95°C for the initial denaturation, 25 cycles of denaturation at 95°C for 30 sec, annealing at 55°C for 30 sec and extension at 72°C for 30 sec, and a final extension step at 72°C for 5 min. PCR products of around 460 bp were purified using a magnetic bead-based system (Agencourt AMPure XP; Beckman Coulter) and sequenced on Illumina MiSeq platform with the 2 × 250 bp paired-end protocol, according to the manufacturer’s guidelines (Illumina). Briefly, each indexed library was prepared by limited-cycle PCR using Nextera technology, and further purified as described above. The libraries were subsequently pooled at equimolar concentrations, denatured with 0.2 N NaOH, and diluted to 6 pM before loading onto the MiSeq flow cell. Sequencing reads were deposited in MG-RAST (project ID, mgp89161).

### Bioinformatics and statistics

Raw sequences were processed using a pipeline that combines PANDAseq [45] and QIIME [46]. The UCLUST software [47] was used to bin high-quality reads into operational taxonomic units (OTUs) at 0.97 similarity threshold through an open-reference strategy. Taxonomy was assigned through the RDP classifier, using the Greengenes database as a reference (release May 2013). Chimera filtering was performed by using ChimeraSlayer [48]. All singleton OTUs were discarded.

16S rRNA gene sequencing data of our cohort were compared with publicly available data from the following previous studies: De Filippis *et al*. [23] (127 Italians; NCBI Sequence Read Archive (SRA) accession number: SRP042234), Schnorr *et al*. [5] (16 Italians and 27 Hadza hunter-gatherers from Tanzania; MG-RAST ID: 7058), Obregon-Tito *et al*. [6] (25 Matses hunter-gatherers from Peru; NCBI SRA: PRJNA268964), and Girard *et al*. [24] (21 Inuit from the Canadian Arctic; Qiita ID: 10439). Genus-level community composition was generated for all cohorts combined. Alpha diversity was assessed using the Shannon and Simpson indices. Beta diversity was evaluated using the Bray-Curtis dissimilarity measure. All statistical analysis was performed in R 3.3.2, using R Studio 1.0.44 and the libraries vegan, made4 and stats. The significance of data separation in the Principal Coordinates Analysis (PCoA) of Bray-Curtis distances was tested using a permutation test with pseudo-*F* ratios (function adonis of vegan package) and ANOSIM test. Superimposition of bacterial genera on the PCoA plot was performed using the envfit function of vegan. Wilcoxon test was used to assess significant differences between groups (for intra- and inter-individual diversity), while Kruskal–Wallis test was used for multiple comparisons. P-values were corrected for false discovery rate (FDR, Benjamini-Hochberg) and P*-*values ≤ 0.05 were considered statistically significant.

## Supporting information

S1 Fig Phylogenetic structure of the gut microbiome of Italian adults adhering to the modern Paleolithic diet. Bar plots of the genus-level composition of the gut microbiome of the enrolled subjects. Only bacterial genera with relative abundance > 0.5% are shown. *, unclassified.
(TIF)

S2 Fig Superimposition of the genus relative abundance on the PCoA plot. Arrows represent the direction of significant correlations (permutation correlation test, P-value < 0.001). A significant segregation among study populations was found (P-value < 1 × 10^−5^; permutation test with pseudo-*F* ratios). MPD = Modern Paleolithic Diet; MD = Mediterranean Diet.
(TIF)

S3 Fig *Prevotella*-*Bacteroides* ratio. Different letters in the boxplots indicate significant differences (P-value < 0.05, Wilcoxon test).
(TIF)

S1 Table Anthropometric data of the enrolled cohort. (XLSX)

S2 Table Modern Paleolithic Diet (MPD) macro- and micro-nutrients summary, based on MétaDieta output (related to Fig 1). Available information on the dietary patterns of the populations considered in the present study (i.e. urban Italians adhering to the Mediterranean diet [23], Hadza from Tanzania [5], Matses from Peru [6], and Inuit from Canadian Arctic [24]) is briefly summarized.
(XLSX)

S3 Table Results of adonis and ANOSIM statistics applied to ordination analysis based on Bray-Curtis dissimilarity index (related to Fig 4A and S2 Fig). (XLSX)

S4 Table Percentage contribution of the main bacterial genera to the community structure based on SIMPER analysis (related to Fig 2). MPD: modern Paleolithic diet; MD: Mediterranean diet; ava: average abundance of group a; avb: average abundance of group b.
(XLSX)
