# Supplementary figures and images for: Gut microbiome response to a modern Paleolithic diet in a Western lifestyle context
Source: PLoS One. 2019 Aug 8;14(8):e0220619. doi: 10.1371/journal.pone.0220619 (PMC6687155; doi:10.1371/journal.pone.0220619)

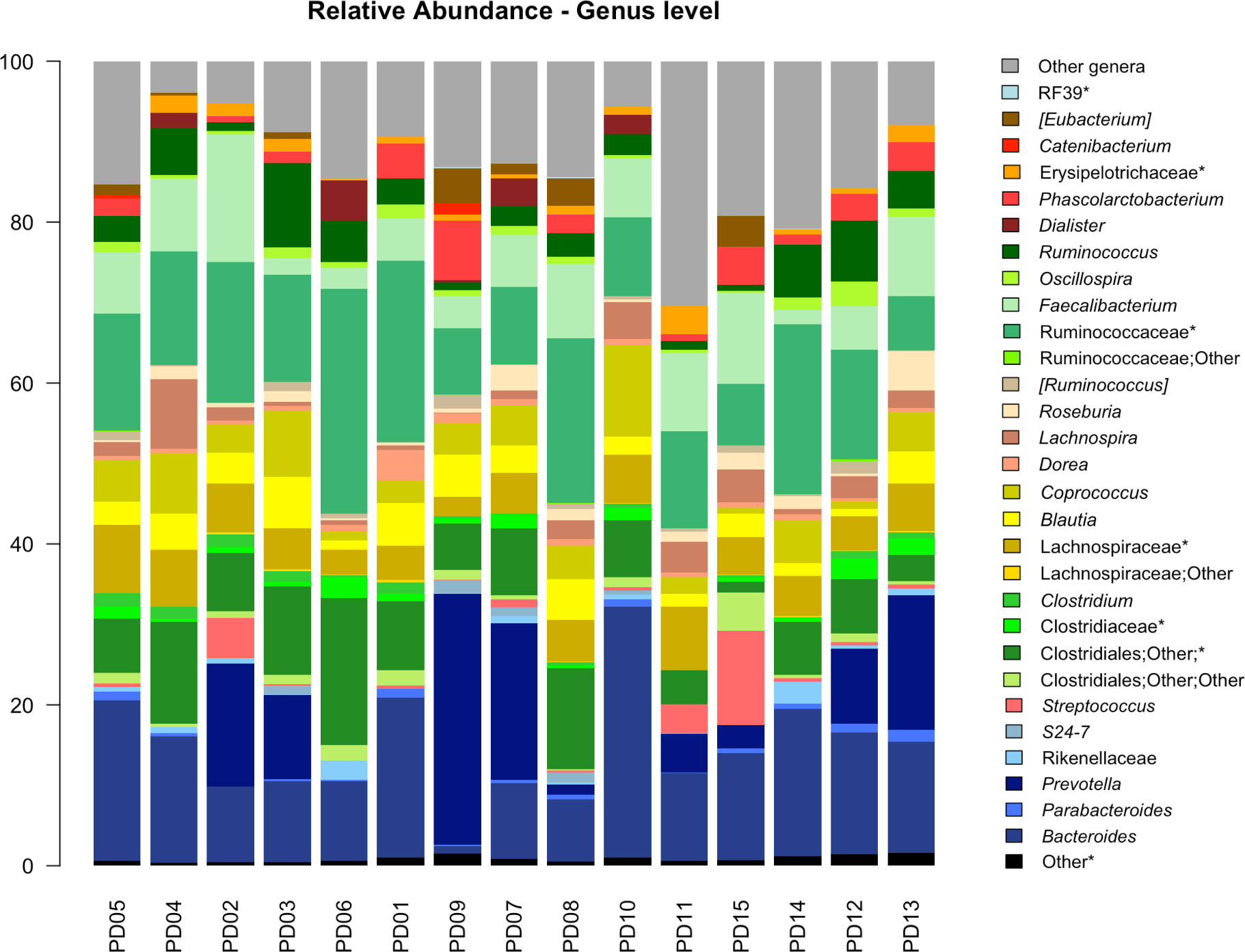

Supplement: S1 Fig — Bar plots of the genus-level composition of the gut microbiome of the enrolled subjects. Only bacterial genera with relative abundance > 0.5% are shown. *, unclassified. (TIF) [file pone.0220619.s001.tif]

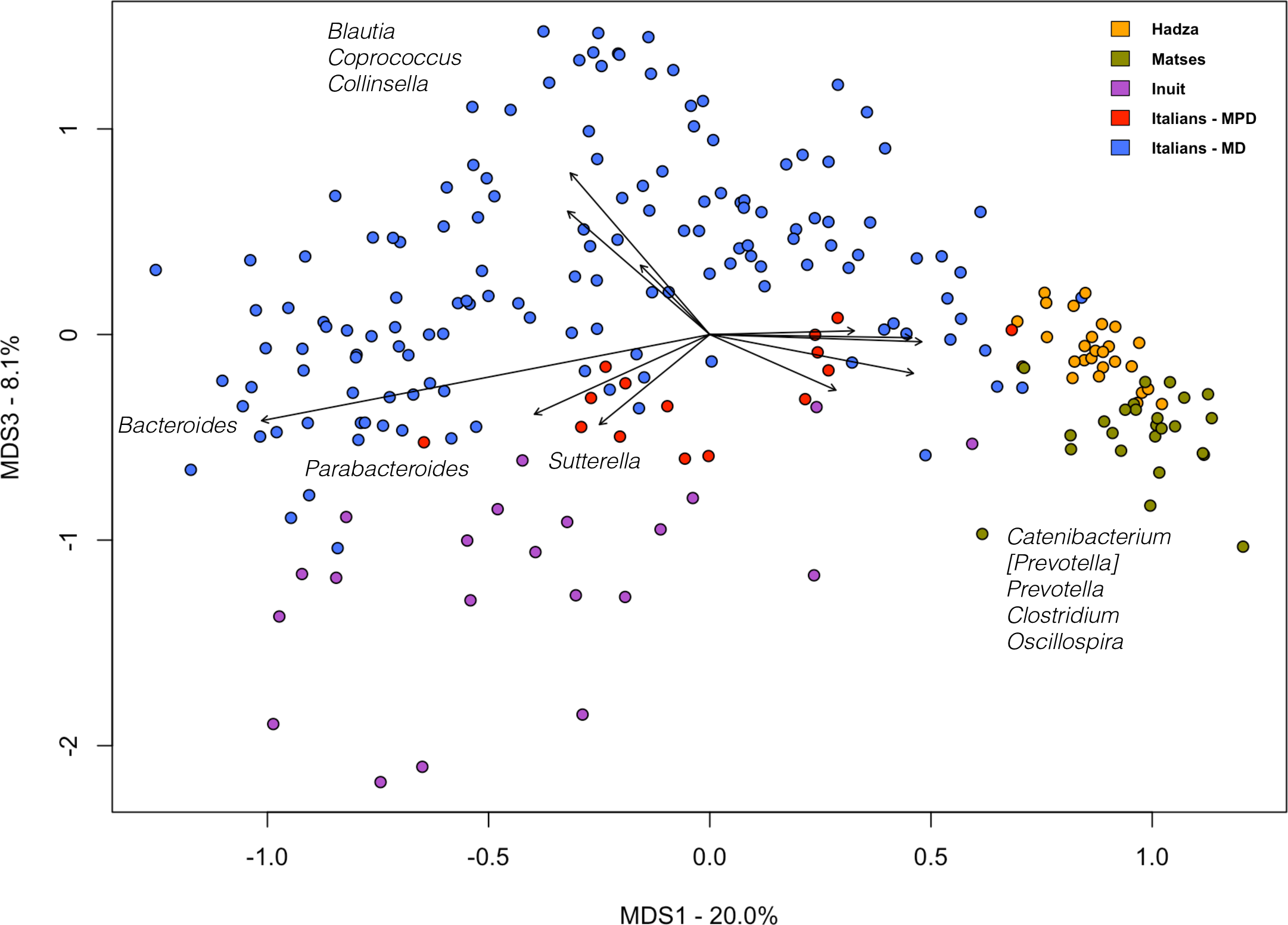

Supplement: S2 Fig — Arrows represent the direction of significant correlations (permutation correlation test, P-value < 0.001). A significant segregation among study populations was found (P-value < 1 × 10^−5^; permutation test with pseudo-F ratios). MPD = Modern Paleolithic Diet; MD = Mediterranean Diet. (TIF) [file pone.0220619.s002.tif]

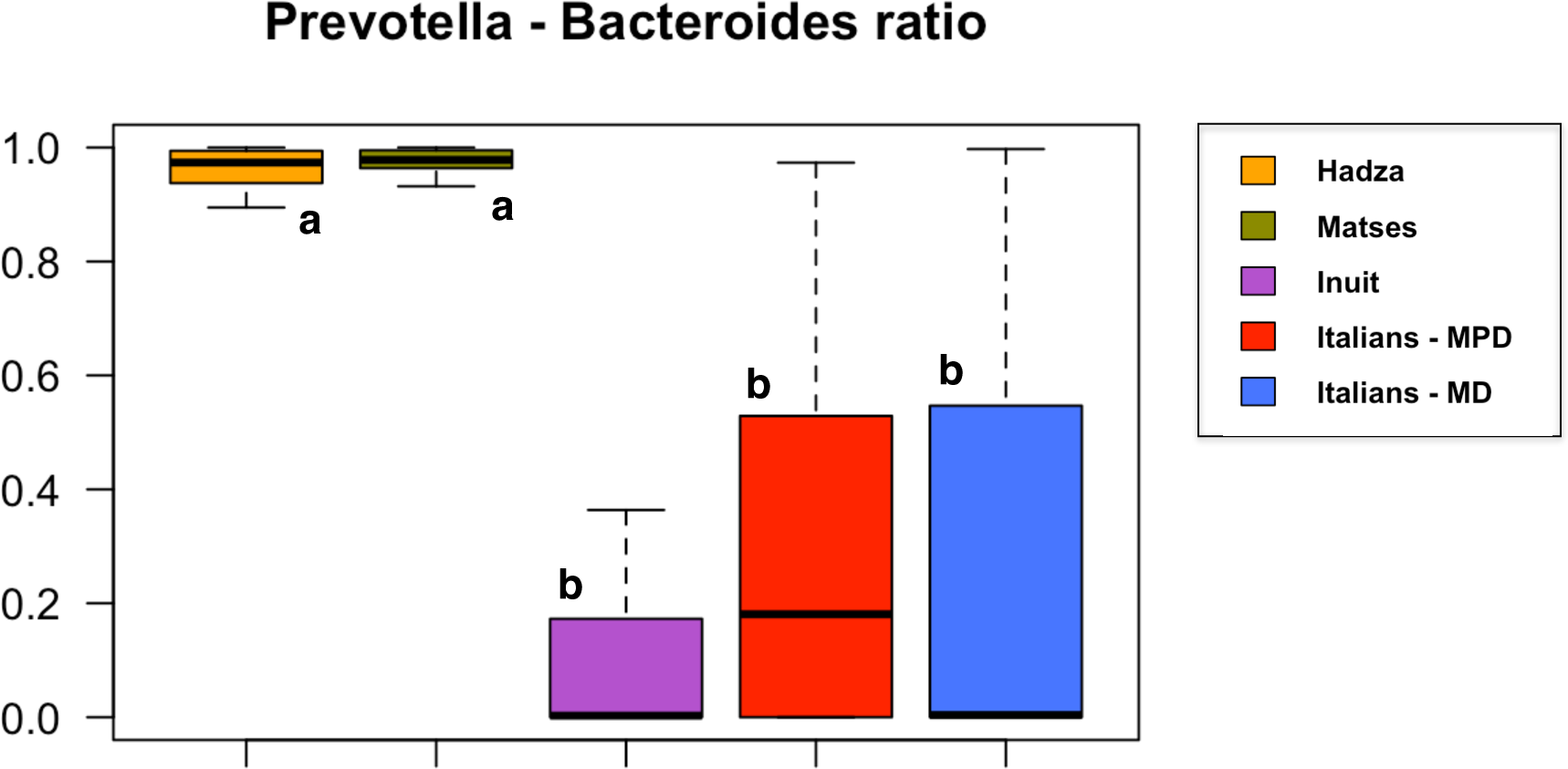

Supplement: S3 Fig — Different letters in the boxplots indicate significant differences (P-value < 0.05, Wilcoxon test). (TIF) [file pone.0220619.s003.tif]
